# 115. The Utility of (1→3)-β-D-glucan Assay in the Diagnosis of Severe Coccidioidomycosis Infections among Immunocompromised Hosts

**DOI:** 10.1093/ofid/ofab466.115

**Published:** 2021-12-04

**Authors:** Parham Ayazi, Tirdad Zangeneh, Aishan Shi, Matthew A Campanella, Mohanad Al Obaidi

**Affiliations:** 1 University of Arizona, Chandler, Arizona; 2 Banner University of Arizona Tucson, Tucson, Arizona; 3 University of Arizona College of Medicine Phoenix, Phoenix, Arizona

## Abstract

**Background:**

Coccidioidomycosis is associated with increased morbidity and mortality in immunocompromised (IC) patients. The diagnosis of invasive fungal infections can be challenging in IC hosts. Culture results may take time to identify *Coccidioides* species, and serologic based tests are less sensitive in IC patients. (1-3)-β-d-glucan (BDG) has been reported to be detected in patients with coccidioidomycosis. We hypothesized that BDG in combination with serology may assist in the early detection of coccidioidomycosis in IC patients.

**Methods:**

After the institutional review board approved the study, we conducted a retrospective chart review from 10/1/2017 through 09/15/2020, including ≥18 years old IC patients with a confirmed diagnosis of coccidioidomycosis by culture. Information regarding demographics, comorbidities, immunosuppression, medications, BDG, serology, and clinical presentation was collected. Patients with infusions that can result in possible false-positive BDG were excluded. Patients with other fungal infections were also excluded. Chi-square test was used to compare categorical variables, Wilcoxon rank-sum and Kruskal-Wallis tests were used to compare non-parametric variables, accordingly.

**Results:**

Over the study period, 269 encounters with positive *Coccidioides spp.* cultures were identified, 78/269 of patients were IC patients, 55/78 were excluded, and 23 cases were included in the final analysis. Among the 23 IC patients, the median age was 64, 43% were female, 74% were White. There were 8 post solid organ transplantation, 7 with a hematological malignancy, and 8 with other types of IC conditions. 19/23 had a pulmonary infection. 4/23 patients died within one month of their encounter. There was no statistical significance difference between positive BDG and serology tests, with 12/23 had positive BDG, and another 12/23 had positive serology. Combined serology and BDG detected 18/23 of the Coccidioidomycosis cases. 17% of the cohort died within the one-month follow-up.

**Conclusion:**

The combined use of BDG assay and *Coccidioides* serology increases the sensitivity of coccidioidomycosis diagnosis to 78% in IC patients. Future prospective studies are needed to further evaluate the utility of serum BDG in diagnosing coccidioidomycosis in IC patients.

**Disclosures:**

**Mohanad Al Obaidi, MD**, **Shionogi Inc.** (Advisor or Review Panel member)

